# Cumulative Antibiogram: A Rapid Method to Hinder Transmission of Resistant Bacteria to Oral Cavity of Newborn Babies

**DOI:** 10.3390/antibiotics12010080

**Published:** 2023-01-02

**Authors:** Mădălina Adriana Malița, Loredana Sabina Cornelia Manolescu, Cristina Florentina Pîrvu, Radu Catalin Costea, Elena Cristina Marcov, Mihai Burlibasa, Daniela Aurelia Pîrvu, Liliana Burlibașa, Mihaela Corina Radu, Irina Prasacu, Viorel Ștefan Perieanu

**Affiliations:** 1 Department of Dental Technology, Faculty of Midwifery and Nursing, “Carol Davila” University of Medicine and Pharmacy, 050474 Bucharest, Romania; 2 Department of Microbiology, Parasitology and Virology, Faculty of Midwifery and Nursing, “Carol Davila” University of Medicine and Pharmacy, 050474 Bucharest, Romania; 3 Department of Scientific Research Methodology-Ergonomics, Faculty of Dentistry, “Carol Davila” University of Medicine and Pharmacy, 0102210 Bucharest, Romania; 4 Department of Restorative Dentistry, Faculty of Dentistry, “Carol Davila” University of Medicine and Pharmacy, 0102210 Bucharest, Romania; 5 Department of Dental Prostheses, Faculty of Dentistry, “Carol Davila” University of Medicine and Pharmacy, 0102210 Bucharest, Romania; 6 Department of Genetics, Faculty of Biology, University of Bucharest, 060101 Bucharest, Romania; 7 Department of Birth, Obstetrics and Gynecology Hospital, 100409 Ploiesti, Romania; 8 Department of Fundamental Sciences, Faculty of Pharmacy, “Carol Davila” University of Medicine and Pharmacy, 050474 Bucharest, Romania

**Keywords:** antibiotic susceptibility testing, cumulative antibiogram, maternal-to-neonate transmission

## Abstract

Background: A rapid bacterial diagnostic is needed more and more in the treatment of patients, because of the emergence of antibiotic resistance. The cumulative antibiogram, an annual report that monitors antimicrobial resistance trends in health care facilities, may provide a profile of empirical therapy useful in diverse emergency situations, such as transmission of resistant bacteria to oral cavity of newborn babies. We aimed to draw a profile of antibiotic resistance encountered. Methods: We assessed the antibiotic resistance (ABR) profile in childbearing women and newborn babies in Ploiesti Obstetrics and Gynecology Hospital by the disk diffusion method characterizing the multidrug-resistant organisms after isolation and identification by phenotypic tests. Extended-spectrum β-lactamase (ESBL)-producing Enterobacterales (ESBL-E), Carbapenem-resistant Enterobacterales (CRE), vancomycin-resistant Enterococci (VRE), methicillin-resistant *Staphylococcus aureus* (MRSA) and vancomycin resistant Group B Streptococcus (VR-GBS) were detected. Results: The prevalence of antibiotic resistance was 11.32% (53/468), while the prevalence of the ESBL-E, MRSA, VRE and VR-GBS strains was 8.34% (39/468). Within the bacteria isolated from fifty-three childbearing women, the prevalence of ESBL-E, MRSA, VRE and VR-GBS was 22.64% (12/53), 32.08% (17/53), 11.32% (6/53) and 7.55% (4/53). In the whole studied group, the prevalence was 2.56% (12/468), 3.63% (17/468), 1.28% (6/468) and 0.86% (4/468). Resistant bacteria were detected at birth in the oral cavity of the newborn babies in all cases. Maternal and neonatal isolates shared similar characteristics. Conclusions: Cumulative antibiogram is useful in case of empiric treatment needed in diverse emergencies, such as transmission of resistant bacteria to oral cavity of newborn babies.

## 1. Introduction

Antibiotic susceptibility testing (AST) is a very important tool that helps clinicians to choose a proper and effective antibiotic therapy and the correct dosage in order to be able to treat patients against bacterial infectious diseases [1,2,3,4]. At the same time, to support clinical decisions, it is imperative to monitor emerging patterns in antibiotic resistance (ABR) at the local level. AST may provide a profile of empirical therapy that may be used in very diverse emergency situations [5]. One field where AST is extremely useful is Obstetric Department, where the cumulative antibiogram report may be literally a life saver. In the Obstetric Departments there are at stake at the same time the lives of two individuals: the childbearing women that are to give birth in emergency as well as their newborn babies [6].

The cumulative antibiogram is an annual report that monitors antimicrobial resistance trends in health care facilities using an annual summary of susceptibility to antibiotic therapies rates [7]. Rapid bacterial diagnostic is needed more and more in the treatment of patients, because of the emergence of antibiotic resistance [8]. In 2022, Clinical and Laboratory Standards Institute (CLSI), published new guideline M39—Analysis and Presentation of Cumulative Antimicrobial Susceptibility Test Data recognizing the need to develop practical but clinically and epidemiologically useful recommendations for the analysis and presentation of data on antimicrobial susceptibility trends [9].

Many resistant bacteria such as extended-spectrum β-lactamase (ESBL)–producing Enterobacterales (ESBL-E), Carbapenem-resistant Enterobacterales (CRE), vancomycin-resistant Enterococci (VRE), methicillin-resistant *Staphylococcus aureus* (MRSA) and vancomycin-resistant Group B *streptococcus* (VR-GBS) were found to give great impact of long-term carriage and development of infection among childbearing women. We will refer at them as extreme resistant bacteria that may impact maternal-to-neonate transmission rates [10]. One way of acquiring ABR bacteria in newborn babies is during vaginal birth, putting the newborn at risk of severe neonatal infections [11,12,13,14]. Infections in newborn babies due to ESBL-producing *E. coli* and MRSA may cause high rates of morbidity and mortality [15,16]. The carriage of ABR bacteria in pregnant women was identified as a risk factor for colonization the oral cavity of the newborn baby [17,18], so, studying the prevalence of ABR bacteria and hindering the transmission from childbearing women to their babies became a must. The colonization levels may differ by country or even region, so a hospital study is more useful when it comes to practical needs [17,18,19]. If data on ESBL-E and MRSA prevalence are to be found, data on CRE, VRE or VR-GBS prevalence in this category are limited.

Here, in the present study, we tried to assess the antibiotic resistance profile of the bacterial strains that were isolated from the childbearing women and transmitted to oral cavity of their newborn babies showing the need of cumulative antibiogram for one clinical obstetrical section in a Ploiesti Obstetrics and Gynecology Hospital (POGH). We aimed to draw a profile of antibiotic resistance encountered in our Obstetrics and Gynecology section. This may be useful to establish a possible empiric treatment recommendation for emergency situations when the time is critical.

## 2. Results

### 2.1. Pregnancy Status, Sociodemographic, and Educational Characteristics, Baseline Clinical Data of Participating 

There were 1932 childbearing women evaluated for diverse presentation problems at the Emergency Room of the Obstetrics and Gynecology Hospital from Ploiesti (collapsed cervix, painful uterine contractions and rupture of membranes) for one year. To all women admitted in hospital bacteriological testing was performed. Most women, 1464 gave birth by Caesarean surgery. The rest, 468 childbearing women, gave birth naturally (vaginal birth) at 12 h from admission to the hospital. The result of bacteriological tests was positive for 223 women, 170 women that gave birth by C-section and 53 women that gave birth naturally. The characteristics of the 170 women that gave birth by C-section and were positive for bacterial culture are presented in Table 1. We identified six different bacteria in the 170 women group, three Gram-negative bacilli: *E. coli, Klebsiella pneumoniae, Proteus* spp. and three Gram-positive cocci: *Staphylococcus aureus*, *Enterococcus* spp., and *Streptococcus agalactiae*. The test for sensitivity came out positive only for antibiotics that are not usually recommended to be administered neither to the woman that just gave birth, nor to her newborn baby: trimethoprim-sulfamethoxazole (SXT), tetracycline (TE), gentamycin (CN), chloramphenicol (C), and amoxicillin/clavulanic acid (AMC). These women that delivered via Caesarean surgery were excluded from the study because they did not transmit any bacteria to their newborn babies. All their newborn babies tested negative for bacteria immediately after birth. 

In the case of 468 women that gave birth naturally, the result of bacterial testing was available only after birth. The result from the bacteriological investigation came out positive for fifty-three women out of 468. All their newborn babies were tested and came out positive as well. The general characteristics of pregnancy status, sociodemographic, educational and clinical data of participating childbearing women that tested positive for bacteria are presented in Table 2. No statistically significative associations were found regarding the area of provenience *p* = 0.5235, level of studies, *p* = 0.8688 or status of pregnancy with the level of resistance, and presence of extreme resistant bacteria or status of membrane at hospital admission. We identified six different bacteria in the studied group, three Gram-negative bacilli: *E. coli, Klebsiella pneumoniae,* and *Proteus* spp. and three Gram-positive cocci: *Staphylococcus aureus, Enterococcus* spp., and *Streptococcus agalactiae.*

We found that the childbearing women that carried bacteria presented the following characteristics: median age 30 years (limits: 17–45); studies: primary school 24.52% (*n* = 13), high school 47.16% (*n* = 25), superior studies 28.3% (*n* = 15); area of residence: rural 56.6% (*n* = 30), and employed 54.72% (*n* = 29) (Table 2).

There were 15.09% (*n* = 8) childbearing women that had four pregnancies or even more, the rest, 84.9% (*n* = 45), had one, two, or three pregnancies. Regarding the number of actual births in our studied group, there were only 7.55% (*n* = 4) childbearing women that had more than three birth; the rest, 92.45% (*n* = 49), had one or two births. The majority, 54.72% (*n* = 29) of the studied women, gave birth between 38 and 39 gestational weeks, 25.53% (*n* = 13) gave birth premature before 37 gestational weeks, and 20.75% (*n* = 11) gave birth after 40 gestational weeks. There were 18.87% (*n* = 10) childbearing women that presented with rupture of the membrane before hospital admission. The rest came with intact membranes at hospital admission. The majority, 79.25% (*n* = 42), of the childbearing women presented ABR bacteria for up to nine antibiotics, while the rest presented bacteria resistant to eleven or even twelve antibiotics. 

### 2.2. Prevalence of ABR among Childbearing Women and Their Newborn Babies

All fifty-three women positive for bacterial culture were carriers of at least one ABR bacteria. There was no childbearing woman positive for more than one strain. The prevalence of ABR in the hole childbearing women group that gave birth naturally by vaginal way was 11.32% (53/468). There were isolated sixteen strains of *Enterobacterales* (nine strains of *E. coli*, three strains of *K. pneumoniae,* and four strains of *Proteus mirabilis*); seventeen *Staphylococcus aureus* MRSA strains; ten *Enterococcus* spp. Strains; and ten *Streptococcus group B* strains. All newborn babies were found positive after birth, with a similar prevalence, 11.01% (52/472); two babies died at birth, and there were twelve pairs of twins. The fifty-three childbearing women and their newborn babies were admitted seven days into the hospital until they were discharged. They were treated with imipenem as follows: the childbearing women were given 500 mg every 8 h, and the newborn babies were given 25 mg/kg every 12 h. Fortunately, no baby developed an infection in the surveillance period that followed hospital discharge.

**Figure 1:**
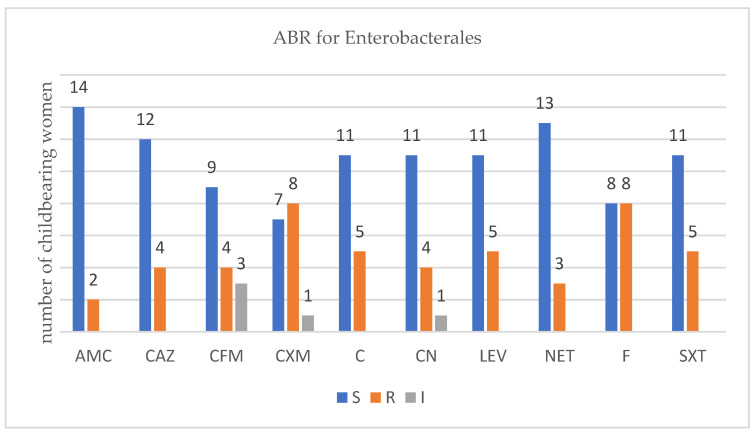
Profile of antibiotic resistance for Enterobactales; S = sensitive, I = intermediate; R = resistant. Legend: amoxicillin/clavulanic acid (AMC), ceftazidime (CAZ), cefixime (CFM), cefuroxime (CXM), chloramphenicol (C), gentamycin (CN), levofloxacin (LEV), netilmicin (NET), nitrofurantoin (F), and trimethoprim-sulfamethoxazole (SXT).

**Figure 2:**
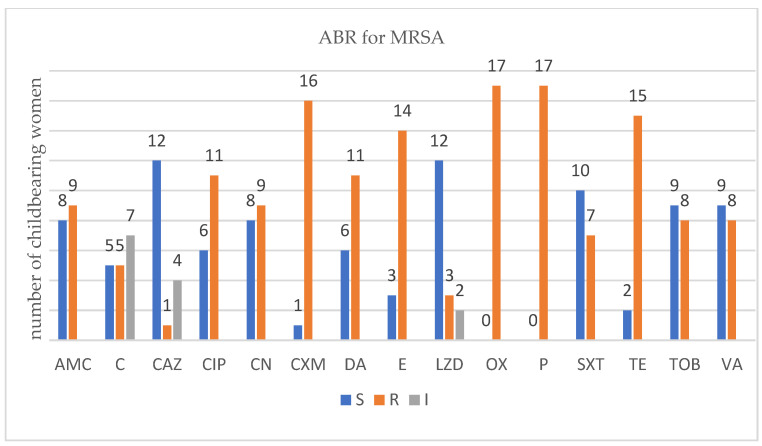
Profile of antibiotic resistance for MRSA; S = sensitive, I = intermediate; R = resistant. Legend: amoxicillin/clavulanic acid (AMC), ceftazidime (CAZ), cefuroxime (CXM), ciprofloxacin (CIP), chloramphenicol (C), clindamycin (DA), erythromycin (E), gentamycin (CN), linezolid (LZD), oxacillin (OX), penicillium (P), trimethoprim-sulfamethoxazole (SXT), tetracycline (TE), tobramycin (TOB), and vancomycin (VA).

**Figure 3:**
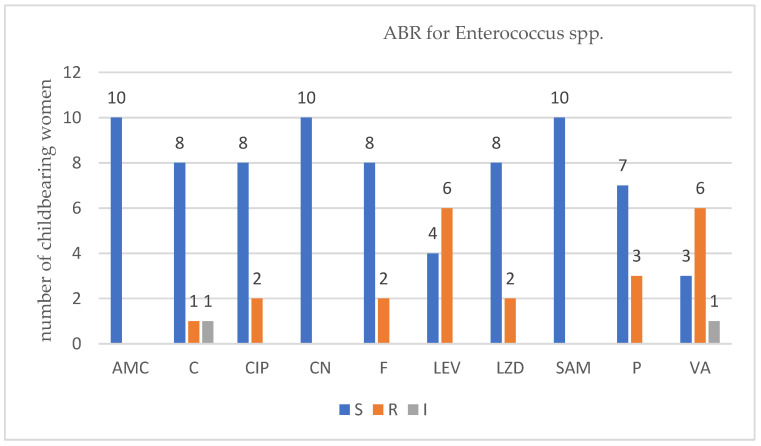
Profile of antibiotic resistance for *Enterococcus* spp.; S = sensitive, I = intermediate; R = resistant. Legend: ampicillin/sulbactam (SAM), amoxicillin/clavulanic acid (AMC), ciprofloxacin (CIP), chloramphenicol (C), gentamycin (CN), levofloxacin (LEV), linezolid (LZD), nitrofurantoin (F), penicillin (P), and vancomycin (VA).

**Figure 4:**
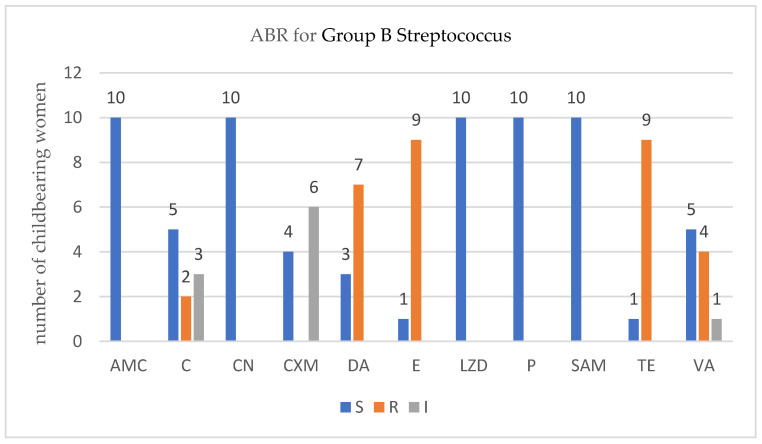
Profile of antibiotic resistance for *Streptococcus group B.*; S = sensitive, I = intermediate; R = resistant. Legend: ampicillin/sulbactam (SAM), amoxicillin/clavulanic acid (AMC), chloramphenicol (C), cefuroxime (CXM), clindamycin (DA), erythromycin (E), gentamycin (CN), linezolid (LZD), penicillin (P), tetracycline (TE), and vancomycin (VA).

For each isolated strain antibiotic resistance testing to several drugs was performed by disk diffusion method (Kirby Bauer) used in accordance with the European Committee on Antimicrobial Susceptibility Testing (EUCAST) 2019 guidelines. Several degrees of ABR were obtained and are presented below for each bacterium type (Figure 1, Figure 2, Figure 3 and Figure 4).

There were sixteen childbearing women that carried three different types of Enterobacterales. Out of them, twelve were carrying β-lactam-producing Enterobacterales (ESBL-E). We found no association between the number of ABR- and the ESBL-producing Enterobacterales*, p* = 0.0667 (Fisher’s exact test). As the antibiotics tested differ for each type of bacterial strains, several figures were designed.

There were 17 childbearing women that carried *MRSA.* All of them displayed several degrees of resistance toward antibiotics. Fisher’s exact test was applied, and a statistically significant association between the number of ABR- and *MRSA*-carrying patients appeared, *p* = 0.0001. MRSA strains exhibited the highest number of antibiotic resistances. The most common resistances encountered were against clinically relevant antibiotics such as oxacillin (all 17 strains), penicillium (all 17 strains), cefuroxime (16 strains), tetracycline (15 strains), erythromycin (14 strains), and, more importantly, a last resort antibiotic such as vancomycin (8 strains).

For *Enterococcus* spp. in the group of ten women that were infected with this type of bacteria, six VRE strains were detected. Applying Fisher’s exact test, no association was found between the number of ABR and VRE*, p* = 0.2090.

In the childbearing women group that were positive for G*roup B Streptococcus*, four VR-GBS strains were detected. Again, no association was found between the number of ABR and the VR-GBS*, p* = 0.2090 (Fisher’s exact test).

### 2.3. Prevalence of Extreme ABR among Childbearing Women and Their Newborn Babies

In the fifty-three group of childbearing women positive for different bacteria with several degrees of resistance toward several antibiotics there were thirty-nine women that carried strain of bacteria of extreme resistance 8.34% (39/468) prevalence, such as twelve β-lactam-producing Enterobacterales (ESBL-E), strains*,* seventeen MRSA strains, six VRE strains, and four VR-SGB strains. The prevalence of ESBL-E, MRSA, VRE, and VR-GBS within the childbearing women group that gave birth naturally was 2.56% (12/468), 3.63% (17/468), 1.28% (6/468), and 0.86% (4/468), respectively. When we analyzed the fifty-three childbearing group the prevalence of ESBL-E, MRSA, VRE, and VR-GBS was high at 22.64% (12/53), 32.08% (17/53), 11.32% (6/53), and 7.55% (4/53). The profile of these women is similar with the general profile already displayed in Table 2.

We found that the childbearing women that carried one bacteria of extreme resistance presented the similar characteristics of the initially studied group without statistically significant differences. Unfortunately, all these carrier mothers delivered the bacteria at birth to their newborn babies. Maternal and neonatal isolates shared similar characteristics. The newborn babies infected with ABR bacteria at birth were followed up for six month and no infection appeared to occur in the respective period. 

## 3. Discussion

The prevalence of antibiotic resistant bacteria has grown and became a threat in several medical domains all over the world [20]. Transmission of these dangerous types of microbes from carrier mothers to their neonates at birth has been documented and discussed [21,22,23,24]. It became clear that vaginal colonization of the childbearing women is a risk factor of transmitting the antibiotic resistant bacteria to the neonate [17,18,25].

### 3.1. Prevalence of ABR in Childbearing Women and Newborn Babies

In our study the prevalence of ESBL-E, MRSA, VRE, and VR-GBS vaginal colonization in the childbearing women group was 2.56% (12/468), 3.63% (17/468), 1.28% (6/468), and 0.86% (4/468). Studies all over the world have reported different rates of childbearing women colonization [11,18,19,26,27,28].

German studies demonstrated that maternal–neonatal transmission of ESBL-E and MRSA from mother to newborn baby is a risk factor for colonization of neonates. They recommended routine screening of neonates and pregnant women as a tool of reducing neonatal morbidity and mortality [11,17]. The prevalence rates of colonization for German childbearing women were similar with our studies for ESBL-E isolates, 2.6% but much lower in case of MRSA isolates (0.5% in German studies compared with 3.63% in our study). Transmission of MRSA to newborn baby was higher in our study as well, 100% versus 53.6%. For ESBL-E we have the same result, 100% transmission rate.

Studies from Israel reported 16% prevalence of cervical colonization higher for ESBL-E isolates, compared with MRSA or VRE isolates, 6% and 1% [17]. The prevalence was lower in Israel study than in our study for maternal-to-neonate transmission, ESBL-E 48%transmittion rate, and MRSA 27.8%, transmission rate. 

In Asia, studies revealed higher levels of ABR; a retrospective study from Korea on childbearing women who underwent emergency cerclage revealed a prevalence of 7.05% (6/85) of VRE isolates, higher compared with 1.28% (6/468) prevalence of VRE from our study and 27.05% (23/85) of ESBL-E colonization of the cervix also higher than in our study 2.56% (12/468); for the Asian newborn babies, their mothers’ colonization was fatal, neonatal death occurring in all cases (100%) [29]. 

In Brazil, macrolide–lincosamide–streptogramin B and tetracycline resistance phenotypes of *Streptococcus group B* spp. were reported, but no vancomycin resistance has been detected [30] compared with our findings of 0.86% (4/468) vancomycin resistant *Streptococcus group B*. 

No carbapenem-resistant Enterobacterales (CRE) were detected in our study. A North America study revealed a CRE prevalence of 2% (2/100) in childbearing [31], while an African study from two maternities showed 4.6% (19/414) and 1.6% (7/422) CRE prevalence in mothers and newborns [23]. It is highly possible that carbapenem antimicrobials are not frequently used to treat Romanian childbearing women, thus explaining our result. We want to underline the fact that in our study the prevalence of ABR bacteria that colonize the cervix of pregnant women was assessed at the moment of delivery and this may differ compared with other studies. The transmission to their newborn babies was established by collecting samples from oral cavity immediate after birth. All tests of the newborn babies turned out positive, and resistant bacteria was transmitted from childbearing probably at the moment of birth, displaying a similar prevalence of 11.01% (52/472) in the newborn babies group.

### 3.2. Cumulative Antibiogram 

Due to these high levels of transmission to neonates found, we considered of great importance the surveillance of the ABR trends displayed at the local level in our case in order to support clinical decision making, infection-control interventions, and of course ABR containment future plans. As to January 2022, The Clinical and Laboratory Standards Institute issued M39-Ed5 a guide that replaced M39-A4 with the title “Analysis and Presentation of Cumulative Antimicrobial Susceptibility Test Data,” intended for clinical laboratories in guidance for preparation of a cumulative antibiogram [9]. In our study we aimed to create such a profile of ABR in childbearing women form one section in a maternity hospital from Romania, where the rates of transmission toward neonates are high. The intended benefit was to have a tool to fight ABR in neonates. When creating a cumulative antibiogram there are some of key points that must be respected and pitfalls that mut be avoided. For example, the data provided of the cumulative antibiogram is intended as a recommendation of empirical therapy of initial infection. Some differences between laboratories practices such as culturing, specimen collection, antimicrobial susceptibility testing or even characteristic of local population may impact the profile of ABR, the trends in susceptibility of the pathogen for a specific drug. A drawback of the result of the cumulative antibiogram is given by testing repeating isolates from the same patient or even limiting the number of tested drugs. 

### 3.3. Oral Cavity Colonization in Newborn Babies

In our study the newborn babies born from carrier mothers were positive and displayed the same strain as the mother, the tested sample being collected from the oral cavity, after natural birth. No statistically significant association was possible for ARB in babies with the presentation of the childbearing women, rupture membranes before hospital admission versus intact membranes at hospital admission, as other studies showed [10,32,33]. Additionally, no antibiotic was used prophylactic during the natural birth or during delivery in the studied childbearing women compared with other studies [34].

Oral cavity colonization with resistant bacteria have been previously documented and it was shown that it poses a clinical threat to further normal development [35], being associated with many dental pathologies, oral mucosal infections that may spread to the respiratory system and trigger life-threatening infections [36,37,38,39,40,41]. There are studies in toddlers that demonstrated how different resistant bacteria are maintained within the indigenous oral microbiota of children, even though they have not been directly exposed to antibiotic therapy [42].

### 3.4. Antibiotic Resistance of the Isolates

Since all isolates have been transmitted to the newborn babies form the childbearing women, we cannot link the level of antibiotic resistance to the level of transmission of resistant bacteria. Unfortunately, the resistance of the newborn babies’ strains was similar to their mothers’ isolates. This is in accordance with the fact that newborns’ bacterial colonization result from birth transmission from the infected childbearing women.

In ESBL-E carriers isolates that came from childbearing women or newborn babies, we noticed an ABR level up to six antibiotics, less than in recent studies published [43]. In MRSA carriers, childbearing women, or newborn babies, we noticed an ABR level up to twelve antibiotics which was higher that in recent studies [10]. In VRE and VR-GBS isolates, childbearing women, or newborn babies, we noticed an ABR level up to four antibiotics which was similar with recent studies [44,45].

### 3.5. Clinical Implications 

In our childbearing women and newborn babies studied group, the most frequent resistant bacteria encountered was MRSA, followed by ESBL-E, VRE, 3.63% (17/468), and VR-GBS. This result must be of public concern, since there is no policy of screening the newborn babies for ABR. The transmission rates from mother to neonate were high but after a six month follow up the outcome was good. It is important to establish maternal colonization prior birth in order to defer neonatal transmission, screening polices, and algorithms of empirical treatments should be developed and implemented.

### 3.6. Strengths and Limitations 

The study is innovative, as the investigation of different types of ABR found in one hospital and transmitted toward neonate 100%, thus showing the importance of having a cumulative antibiogram program. The strengths of the results come from the fact that we followed the rules of laboratory work when assessing the results, we had only one isolate per patient, and we used the same methods for all tests worked for the studied period. The study has several limitations: first, it was performed only on one section of a hospital; second, our group of patients with ABR was small: fifty-three childbearing women, and the group of patients with ESBL-E, MRSA, VRE, and VR-GBS isolates was even smaller: 39 patients. The results may not be generalized for other hospitals but may be used within our department for improving clinical practice. Another drawback may be the fact that we did not consider all the factors that may contribute to transmission of resistant bacteria to the newborn babies. 

## 4. Materials and Methods

### 4.1. Study Population

The study population included 1932 childbearing women, aged ≥ 17, who presented themselves at the emergency room of the Ploiesti Obstetrical and Gynecology Hospital (POGH), Ploiesti, Romania. The childbearing women were clinically evaluated and hospitalized in various sections of the Ploiesti Maternity Hospital depending on the pathology presented (diseases associated with pregnancy, most frequently urinary tract infections, gestational diabetes and threat of premature birth). The 1464 women delivering via Caesarean surgery were excluded from the study. The 468 women evaluated for collapsed cervix, painful uterine contractions, rupture of membranes gave birth by vaginal way 12 h after admission to hospital. 

The standard protocol of the hospital is to test all childbearing women, to collect secretion from the cervix and rectum in pregnant women with intact membranes, or amniotic fluid in the case of pregnant women with ruptured membranes in order to perform a bacteriological examination. The results were out after at least 48 h. Since, in this group of 468 pregnant women, the births took place in the next 12 h after admission, we could not have a result of the bacteriological examination or a recommendation for prophylaxis of the newborn baby right away. The result from the bacteriological investigation was positive in the case of 53 pregnant women that gave birth naturally and 170 women that gave birth by C-section from women that were excluded from the study. All the newborn babies delivered by the positive childbearing women were tested as well.

We decided to exclude the women that delivered by C-section because they could not transmit bacteria to the oral cavity of the newborn babies by birth.

The study was approved by the POGH Ploiesti ethics committee (approval No. 41482/09.08.2022); all the procedures in the study respected the ethical standards of the Helsinki Declaration. Informed consent was compulsory. Each pregnant woman signed a consent form before enrollment. For the newborns’ enrollment in the study, both the mother and the father provided a signed consent.

### 4.2. Sample Collection

Cervical, rectal, and amniotic fluid samples were collected from the childbearing women at the admission using cotton swabs (e-swab, Amies Medium, COPAN, Italy, cat.no.480CE) and were immediately transferred to the laboratory. Oral samples were collected from the newborn babies in the first 48 h after birth (e-swab, Amies Medium, COPAN, Italy, cat.no.480CE). Maternal and neonatal samples were first seeded on four different selective chromogenic agar plates—CHROMID ESBL agar (bioMérieux, Marcy l’Etoile, Lion, France), CHROMID MRSA agar (bioMérieux, Marcy l’Etoile, Lion, France), CHROMagar VRE (CHROMagar, Paris, France), and CHROMagar mSuperCARBA (Paris, France), which were incubated for 24–48 h at 37 °C under aerobic conditions.

Clinical and demographic data were gathered from medical records. The standard protocol is that the women gave birth and they and their newborn babies are hospitalized for three days. In the case of the women that presented positive bacterial cultures, the period of hospitalization was extended during their treatment, along with the treatment of their newborn babies, if there was needed, to seven days. After discharge, a follow-up was done for a period of 6 month by phone call once a month to find out if any infection occurred in the newborn babies that were initially infected with antibiotic-resistant bacteria at birth.

### 4.3. Bacterial Isolation and Identification

After incubation of the samples was performed on agar plates, bacterial isolation followed. All plates with bacterial growth were deferred for bacterial identification. Initially colony morphology (color and size) was observed. Final identification was carried out using Analytical Profile Index, API 20E (for *Enterobacteria*) and API 10 (for *Staphylococcus* and *Streptococcus* genera), biochemical panels from bioMerieux, Paris, France.

We identified six different bacteria in the studied group, three Gram-negative bacilli: *Escherichia coli, Klebsiella pneumoniae,* and *Proteus* spp. and three Gram-positive cocci: *Staphylococcus aureus, Enterococcus* spp., and *Streptococcus agalactiae,* group B streptococcus (GBS).

Bio-typing, based on metabolic characteristics of the strain, was used as a phenotypic typing method to demonstrate the similarities between mother and newborn baby pair strains.

We excluded the source of nosocomial infection or infection associated to medical assistance because collecting of samples, isolation, and identification of bacterial strains were done at hospital admission for the childbearing women. The same is valid in case if the newborn babies, since collecting their samples was performed right away at birth. POGH Ploiesti keeps track of all nosocomial infections monthly and reports them to Public Sanitary Direction of Health as part of a standard protocol. During the evaluated period there were not reported the same bacterial species at the same time, and the incidence of nosocomial infection had a median of 0.45%. Coagulase-negative *Staphylococcus* spp. were detected and reported as nosocomial strains, so we concluded that there was no nosocomial source of contamination during the studied period to our group of childbearing women and newborn babies. Most probably, the colonization of the childbearing women by antibiotic resistant bacteria has a supposed community-associated origin.

### 4.4. Antibiotic Susceptibility Testing

The disk diffusion method (Kirby Bauer) was used in accordance with the European Committee on Antimicrobial Susceptibility Testing (EUCAST) 2019 guidelines to determine the antimicrobial susceptibility of ESBL-producing bacteria and MRSA. The bacterial colonies were dispersed in 0.85% saline to create a 0.5 McFarland standard.

For ESBL isolates, two sets of antibiotics were to be tested. The first one contained amoxicillin/clavulanic acid (AMC), ceftazidime (CAZ), cefixime (CFM), and cefuroxime (CXM) from Sanimed, Romania, and the second one contained chloramphenicol (C), gentamycin (CN), levofloxacin (LEV), netilmicin (NET), nitrofurantoin (F), and trimethoprim–sulfamethoxazole (SXT) from Sanimed, Bucharest, Romania.

For MRSA, the antibiotic used were amoxicillin/clavulanic acid (AMC), ceftazidime (CAZ), cefuroxime (CXM), ciprofloxacin (CIP), chloramphenicol (C), clindamycin (DA), erythromycin (E), gentamycin (CN), linezolid (LZD), oxacillin (OX), penicillin (P), trimethoprim-sulfamethoxazole (SXT), tetracycline (TE), tobramycin (TOB), and vancomycin (VA) from Sanimed, Romania.

For VRE the antibiotic used were ampicillin/sulbactam (SAM), amoxicillin/clavulanic acid (AMC), ciprofloxacin (CIP), chloramphenicol (C), gentamycin (CN), levofloxacin (LEV), linezolid (LZD), nitrofurantoin (F), penicillium (P), and vancomycin (VA) from Sanimed, Romania. 

For VR-GBS the antibiotic used were ampicillin/sulbactam (SAM), amoxicillin/clavulanic acid (AMC), chloramphenicol (C), cefuroxime (CXM), clindamycin (DA), erythromycin (E), gentamycin (CN), linezolid (LZD), penicillium (P), tetracycline (TE), and vancomycin (VA) from Sanimed, Romania.

Susceptibility to vancomycin for VRE and VR-GBS was assessed by the E-test method (bioMérieux, Paris, France). 

Phenotypic typing method such as antibiogram typing (based on the comparison of susceptibility profiles of the pair strains) was used to demonstrate the similarities between mother and newborn baby pair strains.

### 4.5. Characterization of Multidrug-Resistant Organisms (MDRO)

All isolates were examined for the presence of multidrug resistance. The Alifax MDRO screening kits (ALIFAX Polverara (PD), Padova, Italy) were used as phenotypic tests for the effective screening of carries and infected childbearing women and their newborn babies. We carried out the test profile for ESBL/AmpC-producing Enterobacteriaceae, for β-lactam-producing *Enterobacterales* (ESBL-E) (HB&L ESBL/AmpC SCREENING KIT, PD, Italy), the test profile for MRSA screening (HB&L MRSA KIT, PD, Italy), and the test profile for CARBA screening (HB&L CARBAPENEMASE KIT, PD, Italy).

### 4.6. Statistical Analysis 

The data from the questionnaire were analyzed by means of the Microsoft Office package Excel and IBM^®^ SPSS^®^ Statistics Version 23.0 software. For data processing, the COUNTIFS function in Excel was used to filter and sort the initial database. All the categorical characteristics from our study were qualitatively analyzed and are expressed as percentages (%). Pearson’s chi-square (χ^2^) test or Fisher’s exact test was used for small-sized samples to analyze the relationship between the categorical variables. Statistical significance was determined with *p*-value < 0.05.

## 5. Conclusions

ABR isolates transmitted by vaginal birth from childbearing women may be detected in oral cavity of newborn babies. Infection with such resistant bacteria as MRSA, ESBL-E, VRE, and VR-GBS must be treated. In the case of empirical treatment, in an emergency, a cumulative antibiogram may prove to be of real use.

## Figures and Tables

**Table 1:** Pregnancy status, sociodemographic, and educational characteristics, baseline clinical data of 170 childbearing women that tested positive for bacteria and gave birth by C-section.

Characteristic of the Childbearing Women	Carrier of *Enterobactales*	Carrier of *Staphylococcus aureus,*	Carrier of *Enterococcus* spp.	Carrier of *Group B Streptococcus*
**Median age (min–max) years**				
	30 (19–39)	28 (22–41)	29 (17–38)	27 (23–45)
**Studies *n*, (%)**				
Primary school	27, (32.92%)	18, (33.33%)	7, (35%)	5, (35.71%)
High school	27, (32.92%)	18, (33.33%)	7, (35%)	5, (35.71%)
Superior studies	28, (34.14%)	18, (33.33%)	6, (30%)	4, (28.57%)
**Area of provenience *n*, (%)**				
Rural	41, (50%)	28, (51.85%)	18, (90%)	7, (50%)
Urban	41, (50%)	26, (48.18%)	2, (10%)	7, (50%)
**Employed**				
Yes	40, (48.79%)	20, (37.03%)	18, (90%)	7, (50%)
no	42, (51.21%)	34, (62.96%)	2, (10%)	7, (50%)
**Number of pregnancies *n*, (%)**				
≤3 pregnancies ≥4 pregnancies	78, (95.12%) 4, (4.87%)	53, (98.14%) 1, (1.85%)	19, (95%) 1, (5%)	13, (92.85%) 1, (7.14%)
**Number of births (*n*, %)**				
≤2 birth ≥3 birth	82, (100%) 0, (0%)	44, (81.48%) 10, (18.51%)	18, (90%) 2, (10%)	12, (85.71%) 2, (14.28%)
**Gestational week *n*, (%)**				
38–39	82, (100%)	54, (100%)	19, (95%)	14, (100%)
**≥**40	0, (0%)	0, (0%)	1, (50%)	0, (0%)
**Antibiotic resistance (ABR) *n*, (%)**				
≤1ABR ≥5ABR	82, (100%) 0, (0%)	44, (81.48%) 10, (18.51%)	20, (100%) 0, (0%)	14, (100%) 0, (0%)

**Table 2:** Pregnancy status, sociodemographic, and educational characteristics, baseline clinical data of 53 participating childbearing women that tested positive for bacteria and gave birth naturally.

Characteristic of the Childbearing Women	Carrier of *Enterobactales*	Carrier of *Staphylococcus aureus, MRSA*	Carrier of *Enterococcus* spp.	Carrier of *Group B Streptococcus*
**Median age (min–max) years**				
	31.5 (19–39)	29 (22–41)	31 (17–38)	28.5 (23–45)
**Studies *n*, (%)**				
Primary school	5, (31.25%)	6, (35.29%)	2, (20%)	0, (0%)
High school	5, (31.25%)	9, (52.94%)	5, (50%)	6, (60%)
Superior studies	6, (37.5%)	2, (11.76%)	3, (30%)	4, (40%)
**Area of provenience *n*, (%)**				
Rural	8, (50%)	9, (52.94%)	8, (80%)	5, (50%)
Urban	8, (50%)	8, (47.06%)	2, (20%)	5, (50%)
**Employed**				
Yes	10, (62.5%)	7, (41.18%)	7, (70%)	5, (50%)
no	6, (37.5%)	10, (58.82%)	3, (30%)	5, (50%)
**Number of pregnancies *n*, (%)**				
≤3 pregnancies ≥4 pregnancies	13, (81.25%) 3, (18.75%)	14, (82.35%) 3, (17.65%)	9, (90%) 1, (10%)	9, (90%) 1, (10%)
**Number of births (*n*, %)**				
≤2 birth ≥3 birth	12, (75%) 4, (25%)	11, (64.71%) 6, (35.29%)	6, (60%) 4, (40%)	8, (80%) 2, (20%)
**Gestational week *n*, (%)**				
<37	5, (31.25%)	2, (11.76%)	5, (50%)	1, (10%)
38–39	8, (50%)	8, (47.06%)	5, (50%)	8, (80%)
**≥**40	3, (18.75%)	7, (41.18%)	0, (0%)	1, (10%)
**Amniotic fluid port *n*, (%)**				
Rupture of membrane before hospital admission	2, (12.5%)	5, (29.41%)	1, (10%)	2, (20%)
Intact membrane before hospital admission	14, (87.5%)	12, (70.59%)	9, (90%)	8, (80%)
**Antibiotic resistance (ABR) *n*, (%)**				
≤9ABR ≥10ABR	16, (100%) 0, (0%)	10, (58.82%) 7, (41.18%)	10, (100%) 0, (0%)	10, (100%) 0, (0%)
